# Physical activity and frontoparietal network connectivity in traumatic brain injury

**DOI:** 10.1002/brb3.70022

**Published:** 2024-09-18

**Authors:** Emma M. Tinney, Meishan Ai, Goretti España‐Irla, Charles H. Hillman, Timothy P. Morris

**Affiliations:** ^1^ Department of Psychology Northeastern University Boston Massachusetts USA; ^2^ Center for Cognitive & Brain Health Northeastern University Boston Massachusetts USA; ^3^ Department of Physical Therapy, Movement, & Rehabilitation Sciences Northeastern University Boston Massachusetts USA; ^4^ Department of Applied Psychology Northeastern University Boston Massachusetts USA

**Keywords:** frontoparietal control network, physical activity, traumatic brain injury

## Abstract

**Background:**

Prolonged changes to functional network connectivity as a result of a traumatic brain injury (TBI) may relate to long‐term cognitive complaints reported by TBI survivors. No interventions have proven to be effective at treating long‐term cognitive complaints after TBI but physical activity has been shown to promote cognitive function and modulate functional network connectivity in non‐injured adults. Therefore, the objective of this study was to test if physical activity engagement was associated with functional connectivity of the cognitively relevant frontoparietal control network (FPCN) in adults with a TBI history.

**Methods:**

In a case‐control study design, resting state function magnetic resonance imaging and physical activity data from a subset of participants (18–81 years old) from the Cambridge Centre for Ageing and Neuroscience (Cam‐CAN) study was analyzed. Fifty‐seven participants reported a prior head injury with loss of consciousness and 57 age and sex matched controls were selected. Seed‐based functional connectivity analyses were performed using seeds in the dorsolateral prefrontal cortex and the inferior parietal lobule, to test for differences in functional connectivity between groups, associations between physical activity and functional connectivity within TBI as well as differential associations between physical activity and functional connectivity between TBI and controls.

**Results:**

Seed‐based connectivity analyses from the dorsolateral prefrontal cortex showed that those with a history of TBI had decreased positive connectivity between dorsolateral prefrontal cortex and intracalcarine cortex, lingual gyrus, and cerebellum, and increased positive connectivity between dorsolateral prefrontal cortex and cingulate gyrus and frontal pole in the TBI group. Results showed that higher physical activity was positively associated with increased connectivity between the dorsolateral prefrontal cortex and inferior temporal gyrus. Differential associations were observed between groups whereby the strength of the physical activity‐functional connectivity association was different between the inferior parietal lobule and inferior temporal gyrus in TBI compared to controls.

**Discussion:**

Individuals with a history of TBI show functional connectivity alterations of the FPCN. Moreover, engagement in physical activity is associated with functional network connectivity of the FPCN in those with a TBI. These findings are consistent with the evidence that physical activity affects FPCN connectivity in non‐injured adults; however, this effect presents differently in those with a history of TBI.

## Graphical abstract

The objective of this study was to test if physical activity engagement was associated with functional connectivity of the cognitively relevant frontoparietal control network (FPCN) in adults with a traumatic brain injury (TBI) history. Individuals with a history of TBI show functional connectivity alterations of the FPCN. Moreover, engagement in physical activity is associated with functional network connectivity of the FPCN in those with a TBI. These findings are consistent with the evidence that physical activity affects FPCN connectivity in non‐injured adults, however, this effect presents differently in those with a history of TBI.

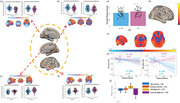

## 1 INTRODUCTION

Traumatic brain injury (TBI) is a prominent neurological disorder, which results in significant emotional, cognitive, and economical burden (Maas et al., 2022). TBIs result from an impact to the head leading to both contact and inertial forces, which initiate a cascade of secondary injury mechanisms affecting molecular, cellular, and network processes (Naumenko et al., 2023). Changes in functional connectivity, the temporal covariation of the blood‐oxygen‐level‐dependent (BOLD) signal (Sharp et al., 2014), have been reported acutely after injury and are primary driver of patient‐reported symptoms and cognitive deficits post‐TBI (Morelli et al., 2021; Puig et al., 2020). While some individuals may recover from this dysfunction, others exhibit persistent (over the first year post‐injury) alterations in functional connectivity in large‐scale networks such as the frontoparietal control network (FPCN; Churchill et al., 2017; DiFabio et al., 2022; Palacios et al., 2017). Yet less is known about how TBI‐induced changes in functional connectivity can persist in the years following injury. Moreover, no efficacious interventions exist to treat the negative effects of TBI, either in the acute or chronic phases of injury. Healthy lifestyle habits, however, such as physical activity, may provide a sustainable and long‐term approach to maintaining brain health in those with a history of TBI.

Physical exercise and physical activity‐based interventions have been shown to improve cognitive function and modulate functional network connectivity in non‐injured populations by promoting changes to cellular and molecular mechanisms (Yu et al., 2021). Notably, physical exercise can induce neuroplasticity (de Sousa Fernandes et al., 2020), leading to changes in large‐scale cortical networks (Moore et al., 2022), including alterations in functional connectivity within the FPCN in older adults (Voss et al., 2010). Similarly, functional connectivity of the FPCN has been shown to support adherence to physical exercise interventions in older adults (Morris et al., 2022). Physical activity engagement seems to be increasingly important for those with a prior TBI, with work demonstrating that those with a TBI have a greater association between physical activity and global and cognitive health (Morris et al., 2019). Moreover, aerobic exercise interventions have been shown to improve functional neuroimaging outcomes in post‐concussion syndrome (Leddy et al., 2013; Sharma et al., 2020). However, prior work had very small sample sizes (*n* = 4 per group), a small age range, only studied acute and sub‐acute concussions, and only studied the effect of a supervised intervention on acute sport‐related concussion. No population‐based studies have studied if voluntary physical activity engagement can affect functional brain network outcomes regardless of age in community‐dwelling individuals with a TBI history. Long‐term adherence to this healthy lifestyle behavior is likely important for global health outcomes (Morris et al., 2019) in TBI, yet more evidence of its effect on objective brain outcomes is necessary to promote this behavior in those living with a TBI.

Physical activity engagement is thought to be supported by executive functioning (Best et al., 2014; Cheval et al., 2020; Daly et al., 2015; Savikangas et al., 2021), highlighted in the dual processing theory (Brand & Ekkekakis, 2018) and temporal self‐regulation theory (Hall & Fong, 2015). Executive functioning includes set‐switching, monitoring and updating information, and inhibiting prepotent responses (Miyake et al., 2000), allowing individuals to take top‐down control of behaviors and self‐regulate (Hofmann et al., 2012). Moreover, executive functioning can aid in suspending default habits (sedentary behavior) and engaging in healthy lifestyle behaviors with effort (physical activity) (Buckley et al., 2014; Hall & Fong, 2015). Evidence shows that the FPCN includes regions identified as supporting cognitive control and decision‐making processes, including the dorsolateral prefrontal cortex, anterior cingulate cortex, and inferior parietal lobule (Dosenbach et al., 2008; Seeley et al., 2007; Vincent et al., 2008). Specifically, we focused on the inferior parietal lobule and the dorsolateral prefrontal cortex. The inferior parietal lobule and the dorsolateral prefrontal cortex have distinct, complementary roles that allow the FPCN to support a range of executive functions and attentional processes, ensuring that behavior is goal directed and adaptable. The inferior parietal lobule is responsible for attention, spatial awareness, and coordination of actions (Andersen & Cui, 2009; Culham & Kanwisher, 2001). The dorsolateral prefrontal cortex is responsible for executive functioning, goal‐directed movement, and adaptive behaviors (Mansouri et al., 2009; Miller & Cohen, 2001; Tanji & Hoshi, 2008). Moreover, the inferior parietal lobule and dorsolateral prefrontal cortex have both been implicated in TBI (Mayer et al., 2011), specifically with the execution of movement (Gooijers et al., 2016). Therefore, the dorsolateral prefrontal cortex and inferior parietal lobe are key regions to the FPCN to study top‐down, control‐type behaviors such as engagement in physical activity.

This current study first aimed to test differences between FPCN functional connectivity in those with a prior TBI compared to age‐ and sex‐matched controls. Second, the study aimed to test if there was an association between physical activity and FPCN functional connectivity in those with a prior head injury. To determine if this was specific to those with a history of TBI, the study aimed to test if there was a differential relationship between physical activity and FPCN functional connectivity in TBI and matched controls.

## 2 METHODS

### 2.1 Participants

This is a case‐control study using a subset of participants from a population‐based study of 708 participants recruited as a part of the Cambridge Centre for Ageing and Neuroscience (Cam‐CAN) study (data used in this work were available at: https://www.mrc‐cbu.cam.ac.uk/datasets/camcan/) (Taylor et al., 2017). See Shafto et al. (2014) for more details on the primary study (Shafto et al., 2014). The ethical approval for the study was obtained from the Cambridgeshire 2 Research Ethics Committee. Participants gave written informed consent. Out of the 708 participants, 63 answered “yes” to the question “Have you ever had a serious head injury and been unconscious after it? (Have you ever been knocked out?).” If they answered “yes,” participants also provided the age at which their injury occurred. Of those 63 participants, 57 had complete data across all variables used in this analysis. Note that age 57 years (range = 19–81 years old), sex (65% female), and education (60% with a degree) randomly matched controls using the *MatchIt* package in R were included in the analysis for a total of 114 participants. Full sample characteristics are shown in Table 1.

**TABLE 1:** Participant demographics.

	Controls (matched) TBI (*n* = 57)	TBI (*n* = 57)	Overall (*n* = 144)
Sex	20 F, 37 M	20 F, 37 M	40 F, 74 M
Age (mean, SD, and range)	55.4 (18.9) [19.2, 81.8]	55.4 (18.8) [19.2, 81.7]	55.4 (18.8) [19.2, 81.8]
**Education**			
A–level	12	12	24
Degree	37	32	69
GCSE/O–level	8	7	15
None	0	6	6
**Physical activity** (mean, SD, and range)	42.0 (20.1) [13.1, 112]	47.1 (29.6) [4.92, 177]	44.6 (25.4) [4.92, 177]
**Years since TBI** (mean, SD, and range)		29.6 (19.4) [0, 69]	
**Mean Head motion** (mean, SD, and range)	0.32 (0.15) [0.14, 0.83]	0.33 (0.15) [0.13, 0.85]	0.33 (0.15) [0.13, 0. 85]

### 2.2 Physical activity

Physical activity energy expenditure (physical activity) was gathered as part of a larger self‐completed questionnaire. The questions about physical activity were based on the European Prospective Investigation into Cancer Study—Norfolk Physical Activity (Wareham et al., 2002). Individual total physical activity per day (kJ/day/kg) was calculated from self‐reported activities into metabolic equivalents, based on the standard definition of 1 MET as 3.5 mL O_2_/min/kg (or 71 J/min/kg) based on the resting metabolic rate (Henry, 2005). Physical activities probed in this questionnaire encompass any physical activity performed at work, at home, during leisure, and commuting typically over the past 12 months. The full questionnaire is found in the supplementary materials.

### 2.3 MRI data acquisition and preprocessing

Structural T1w and resting state functional magnetic resonance images were obtained with a MAGNETOM Trio, A Tim Systems 3T Siemens scanner with a 32‐channel head coil. An 8 min and 40 s resting state scan was acquired with a total of 261 volumes. T1‐weight structural data were acquired by a Magnetization Prepared Rapid Gradient Echo Imaging (MPRAGE) sequence. The parameters were as follows: Repetition time (TR) = 2250 ms, echo time (TE) = 2.99 ms, flip angle = 9°, field of view (FOV) = 256 × 240 × 192, voxel size = 1 × 1 × 1 mm, GRAP acceleration factor = 2. The resting‐state functional images were collected by an echo‐planar imaging sequence. The parameters were as follows: TR = 1970 ms, TE = 30 ms, flip angle = 78°, FOV = 192 × 192, voxel size = 3 × 3 × 4.44, volumes = 261, slices = 32, order = descending. A full protocol with more details is provided in prior Cam‐CAN publications (Shafto et al., 2014; Taylor et al., 2017), with the initial preprocessing performed by the Cam‐CAN team. Anatomical data were normalized into standard Montreal Neurological Institute (MNI) space, segmented into grey matter, white matter, and cerebral spinal fluid (CSF) tissue classes, and warped into a population‐based template through DARTEL procedure (Taylor et al., 2017). Functional images were unwarped using field maps, motion corrected, slice timing corrected, co‐registered to T1 images, and normalized into MNI space. Resting‐state images were further preprocessed using CONN to identify and remove scanner and physiological artifacts (Whitfield‐Gabrieli & Nieto‐Castanon, 2012). Functional data were smoothed with 6‐mm kernel and denoised using a standard denoising pipeline (Nieto‐Castanon, 2020), including wavelet despiking, regression of potential confounding effects characterized by white matter, CSF, six motion parameters, linear trends, followed by bandpass frequency filtering [0.01, Inf] (Geerligs et al., 2017) of the BOLD time series. CompCor (Behzadi et al., 2007) noise components within white matter and CSF were estimated by computing the average BOLD signal as well as the largest principal components orthogonal to the BOLD average, and motion parameters within each eroded segmentation mask. The mean motion was calculated as the scan‐to‐scan average framewise displacement (FD) for each participant based on the six motion parameters (Power et al., 2012). No participant was removed from this analysis for excessive head motion of >0.9 mm mean motion.

### 2.4 Statistical analysis

**FIGURE 1:**
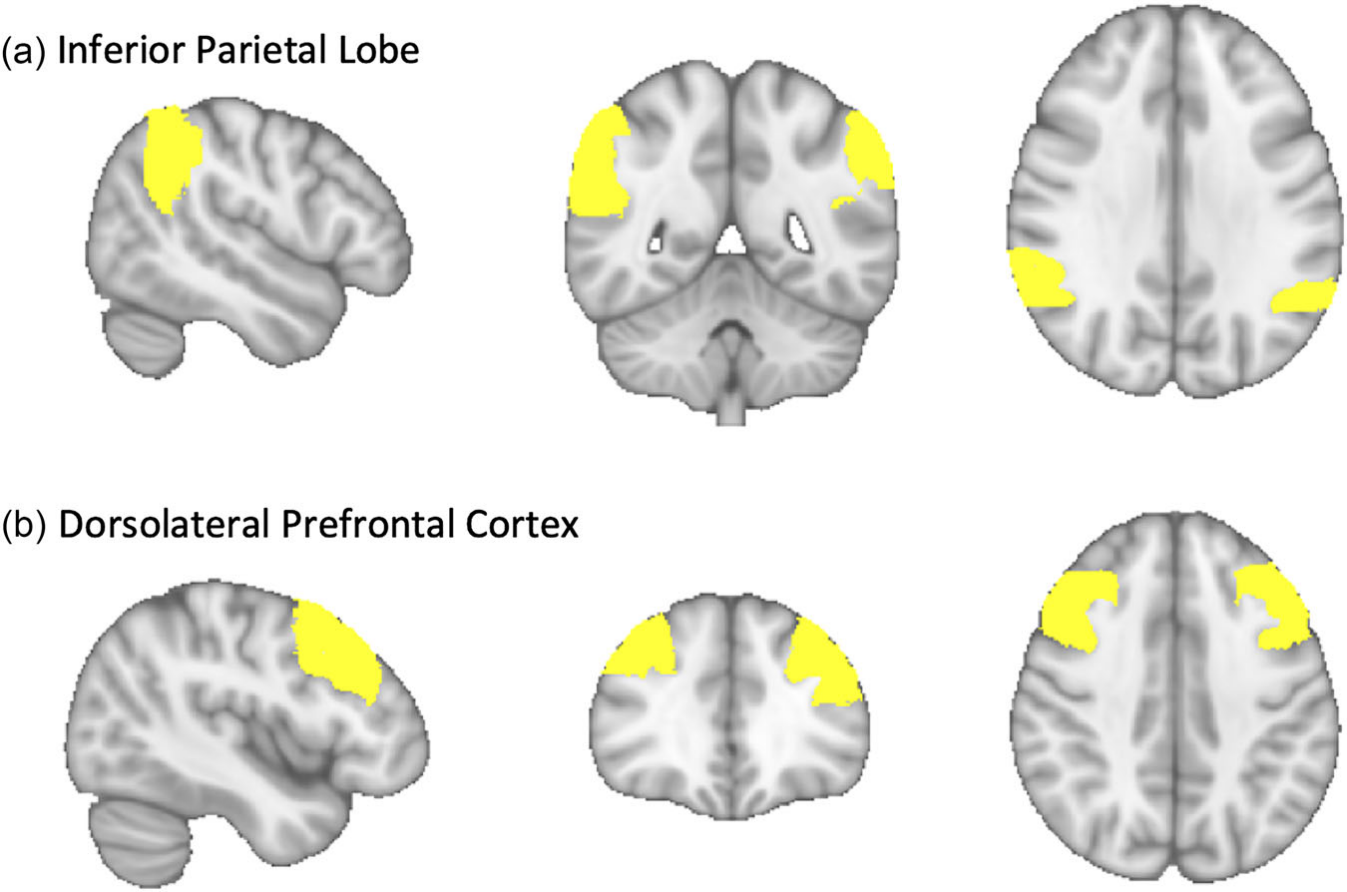
Regions of Interest for seed‐based correlations (SBC) analysis in frontal parietal control network, calculated by overlaying Schaefer atlas overtop Harvard Oxford Atlas anatomical space. (a) Inferior parietal lobe and (b) dorsolateral prefrontal cortex.

Group‐level functional connectivity analyses were performed using seed‐based correlations (SBC) within a General Linear Model framework (GLM) using the CONN toolbox (Nieto‐Castanon, 2020). All other statistical analyses were performed using R (Version 4.3.1) and Rstudio (Version 2023.12.0). Seed‐based connectivity maps (SBC) were estimated characterizing the patterns of functional connectivity with two regions of interest (ROI) (bilateral dorsolateral prefrontal cortex and bilateral inferior parietal lobule; Figure 1). ROIs were calculated by overlaying the Shaefer 100 functional atlas overtop the Harvard Oxford Atlas anatomical space (Morris et al., 2022). The average time series from each seed ROI was extracted. Then, Pearson's correlation coefficients were computed between the averaged time series within each seed and the time series in all other voxels of the whole brain and converted to normalized z‐scores using Fisher transformation prior to performing second‐level analyses. For all analyses, we used a GLM *F*‐test with bilateral seeds jointly entered at the same time and controlling for age, sex, education, mean head motion, and years since injury. Bilateral seeds were entered jointly in the same *F*‐test, a default pipeline in CONN, to reduce the number of comparisons, evaluating any effect among either side. A height‐level statistical threshold of *p* < .001, cluster threshold of *p* < .05 family‐wise error (FWE) corrected, and *k* > 50 were used to determine significant clusters. Three separate *F*‐tests were conducted for each pair of bilateral ROIs; First, we tested if there were differences in functional connectivity in those with a history of TBI versus matched controls. Second, we tested if there was an association between physical activity and functional connectivity in just the TBI group. Third, we tested if there was a differential association between functional connectivity and physical activity in TBI and matched controls. Then, we used the “interactions” (Long, 2021) package in R to calculate simple slopes of the relationship between functional connectivity and physical activity in each group.

We tested between group differences with an analysis of covariance while controlling for age, sex, education, and years since injury. Model assumptions were checked using *Q–Q* residual versus fitted plots to assume dichotomy of outcome variables and linearity. We first tested for differences in mean head motion. We then tested for differences in physical activity between TBI and matched controls.

## 3 RESULTS

### 3.1 Functional connectivity in TBI versus controls

Results revealed no significant differences in mean head motion (*F* = .01, *p* = .91) (Table 1). No participants were removed due to excessive head motion.

**FIGURE 2:**
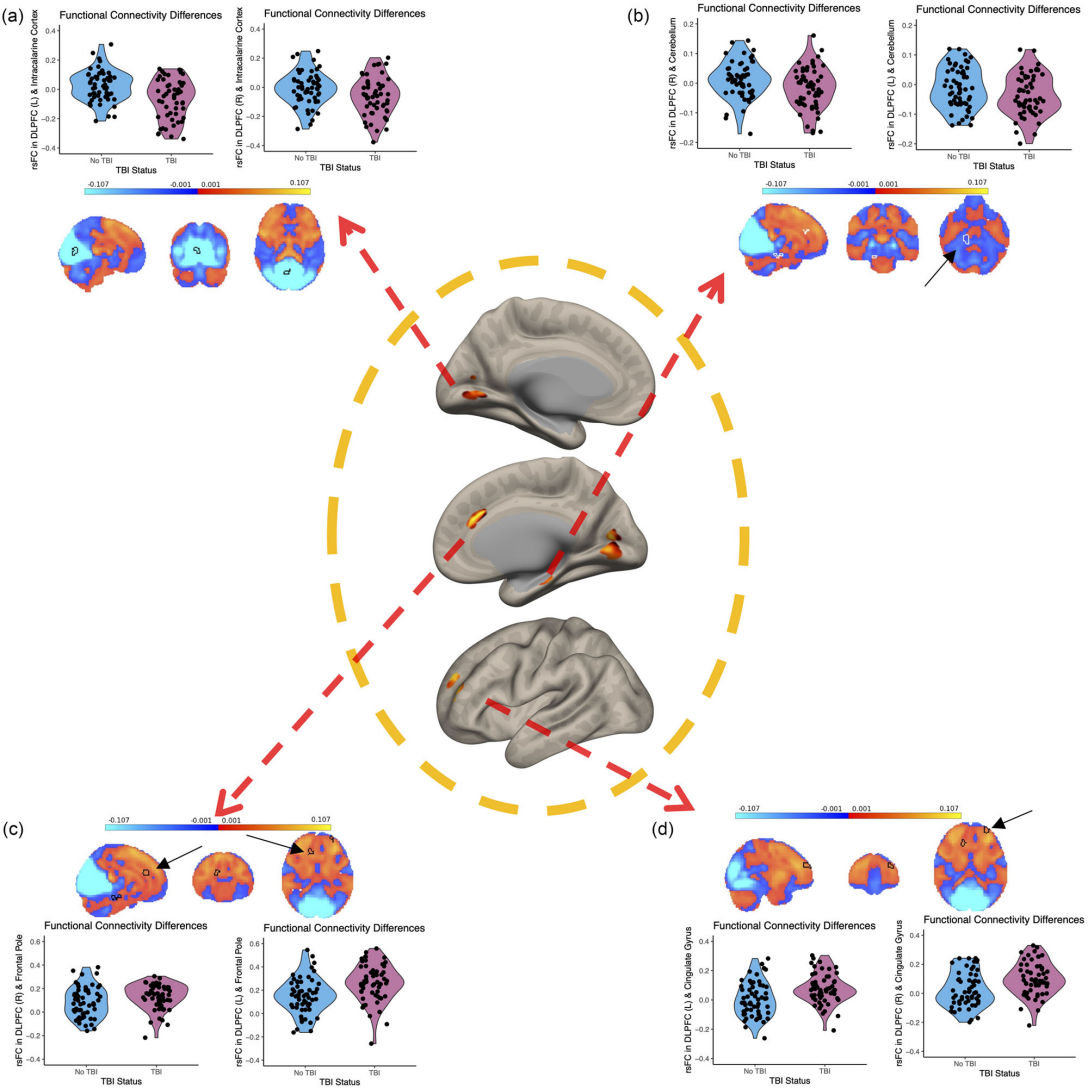
Resting state seed‐based connectivity (rsFC) in the dorsolateral prefrontal cortex (dlpfc) between traumatic brain injury (TBI) and no TBI. Violin plots are demonstrating the connectivity strength between dorsolateral prefrontal cortex and four resulting clusters in both groups and the transparent threshold map of connectivity correlations (*r* = −.1–.1) for this analysis with the significant clusters outlined in black. (a) Cluster within the intracalcarine cortex with lower connectivity in the TBI group. (b) Cluster spanning the cerebellum with lower connectivity in the TBI group. (c) Cluster within the frontal pole higher connectivity in the TBI group. (d) Cluster within cingulate gyrus with higher connectivity in the TBI group.

To test for differences in the group of individuals who reported having a prior head injury, seed to voxel results from the dorsolateral prefrontal cortex revealed four clusters that had significantly different functional connectivity between those with a history of TBI versus matched controls (Figure 2). All clusters are voxel *p* < .001 uncorrected, cluster *p*‐FWE < .05. Full statistical information and coordinates are shown in Table 2. A cluster within the cingulate gyrus showed increased connectivity in TBI with both the right and left dorsolateral prefrontal cortex (Figure 2a). A cluster spanned the intracalcarine cortex and lingual gyrus and showed decreased connectivity in TBI with both the right and left dorsolateral prefrontal cortex (Figure 2b). A cluster sitting within the frontal pole showed increased connectivity in TBI with both the right and left dorsolateral prefrontal cortex (Figure 2c). A cluster sitting within the cerebellum showed decreased connectivity in TBI with both the right and left dorsolateral prefrontal cortex (Figure 2d). Functional connectivity of the inferior parietal lobule was not significantly different between groups.

**TABLE 2:** Summary of functional connectivity results from all models.

Cluster	Seed	Label	*X*	*Y*	*Z*	Cluster size	Size *p*‐FWE	Peak *p*‐FWE	Direction
	TBI versus matched controls								
Cluster 1	Dorsolateral prefrontal cortex	Intracalcarine cortex	+10	−70	+14	260	.002	.90	↓functional connectivity in TBI
Cluster 2	Dorsolateral prefrontal cortex	Cerebellum	+18	−30	−24	190	.01	.46	↓functional connectivity in TBI
Cluster 3	Dorsolateral prefrontal cortex	Frontal pole	−30	+54	+24	141	.04	.43	↑functional connectivity in TBI
Cluster 4	Dorsolateral prefrontal cortex	Cingulate gyrus	+16	+30	+20	122	.07	.53	↑functional connectivity in TBI
	The association between functional connectivity and physical activity in TBI								
Cluster 1	Dorsolateral prefrontal cortex	Inferior temporal gyrus	+52	−18	−36	164	.02	.80	↑ functional connectivity → ↑physical activity
	Physical activity in TBI versus controls								
Cluster 1	Inferior parietal lobule	Inferior temporal gyrus	+58	−60	−16	176	.04	.17	↑ functional connectivity → ↑physical activity in TBI, ↓functional connectivity → ↑physical activity in controls

Abbreviation: TBI, traumatic brain injury.

### 3.2 The association between functional connectivity and physical activity in TBI

**FIGURE 3:**
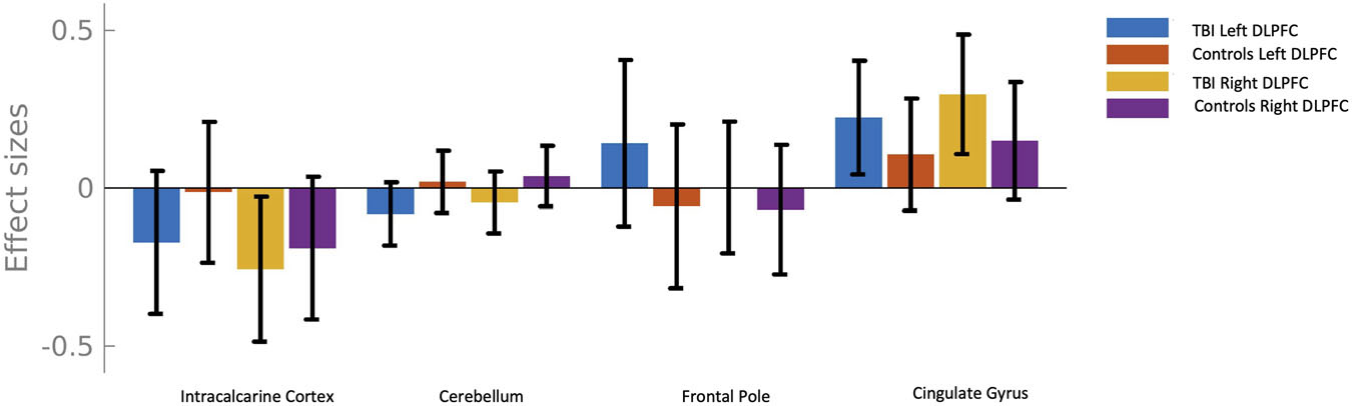
Plot showing effect sizes of each bilateral seed of the dorsolateral prefrontal cortex (DLPFC) and its associated cluster. Negative effect sizes indicate hypoconnectivity and positive effect sizes indicate hyperconnectivity.

**FIGURE 4:**
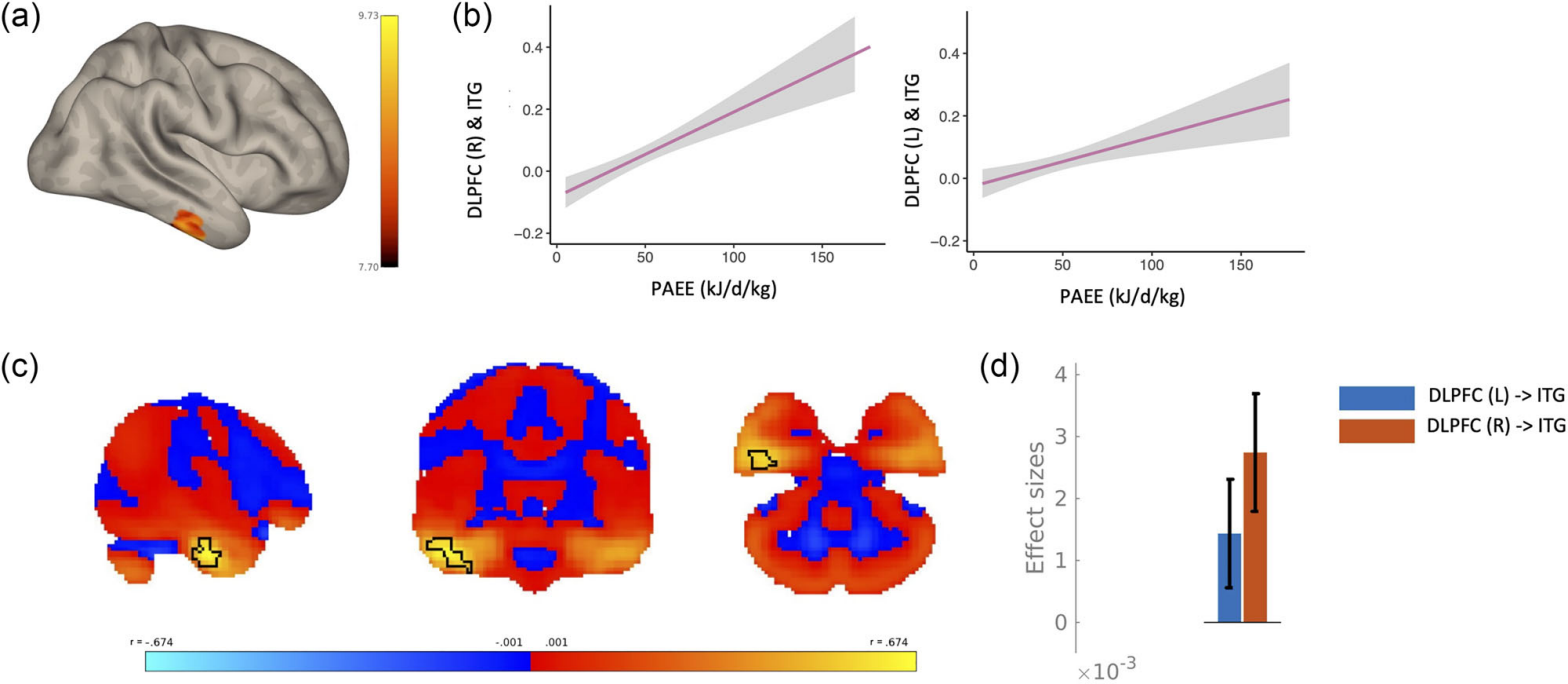
Seed‐based connectivity with associations between physical activity and functional connectivity in traumatic brain injury (TBI) group. (a,b) Higher physical activity (PAEE) was significantly and positively associated with functional connectivity between both the L and R dorsolateral prefrontal cortex (DLPFC) and a cluster in the R inferior temporal gyrus (ITG). (c) Unthresheld map of connectivity correlations. Significant cluster outlined in black. (d) Effect size plot demonstrating the effect sizes of each bilateral seed.

Seed to voxel results from the dorsolateral prefrontal cortex revealed one cluster that was associated with physical activity in TBI (Voxel *p* < .001 uncorrected, Cluster *p*‐FWE < .05) sitting within the inferior temporal gyrus, showing increased connectivity is associated with increased physical activity (Figures 3a and 4). Full statistical information and coordinates are shown in Table 2. Seed to voxel results from the inferior parietal lobule revealed no clusters that were associated with physical activity in TBI.

### 3.3 Physical activity in TBI versus Controls

**FIGURE 5:**
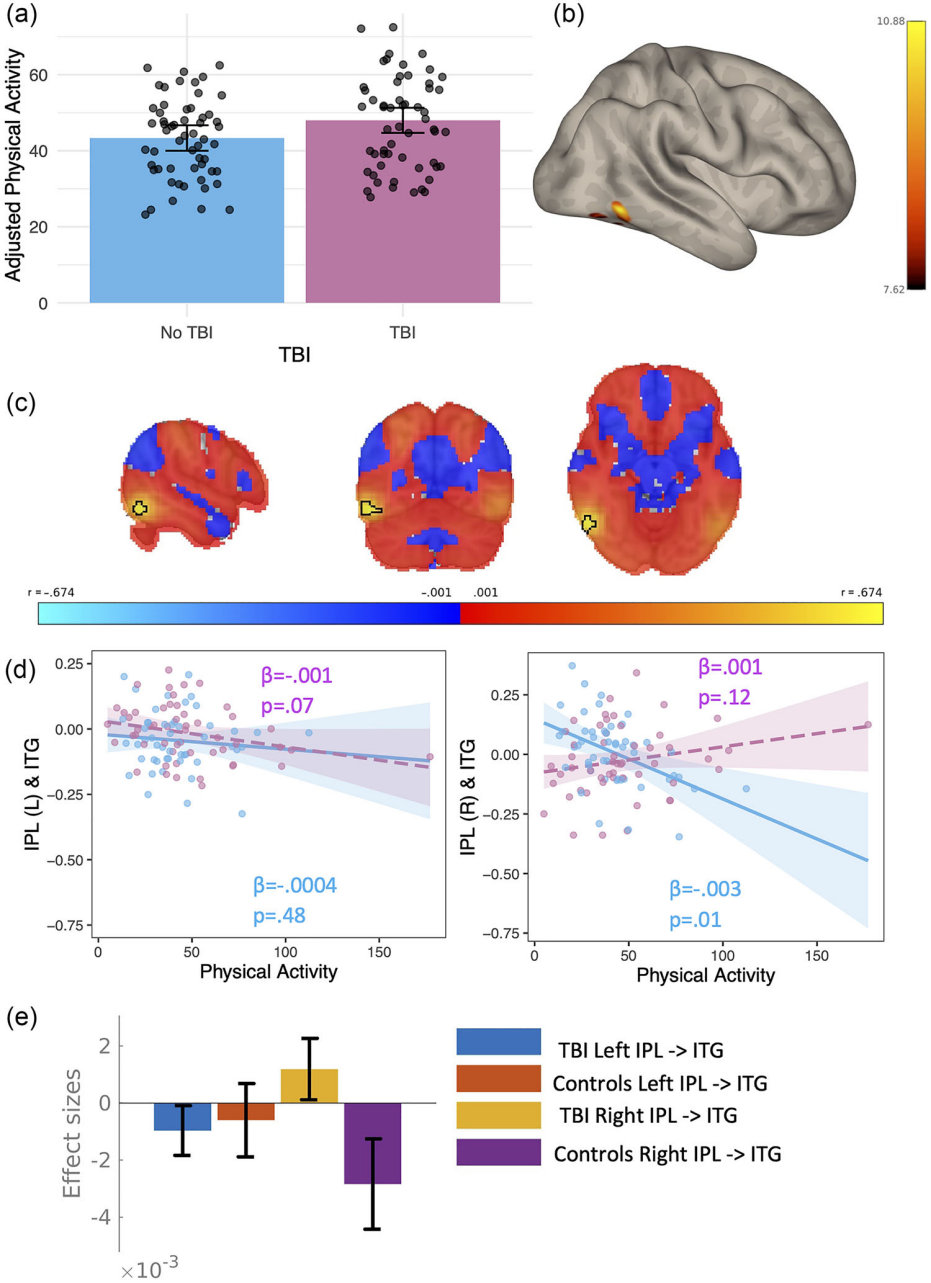
No traumatic brain injury (TBI) and TBI differences in physical activity (a) violin plot showing physical activity in each group. (b) Seed‐based connectivity results from the L and R inferior parietal lobule (IPL) showing the differential relationship between physical activity (PAEE) and TBI and controls in a cluster in the R inferior temporal gyrus (ITG). (c) Thresholded (*r* = .01) map of connectivity correlation strength with statistically significant cluster outlined in black. (d) interaction plot depicting the differential association between physical activity and functional connectivity in TBI (purple) compared to controls (blue). (e) Effect size plot demonstrating the effect sizes of each bilateral seed for each group.

There were no differences between physical activity and TBI status (*F* = 1.23, *p* = .27) (Figure 5a).

Seed to voxel results from the dorsolateral prefrontal cortex from the interaction between physical activity in TBI and physical activity in controls revealed no clusters that had significant differential connectivity between groups. Seed to voxel results from the inferior parietal lobule from the interaction between physical activity in TBI and physical activity in controls revealed one cluster that had significant differential connectivity between groups sitting within the inferior temporal gyrus (Figure 5b). Full statistical information and coordinates are shown in Table 2. The TBI group demonstrated a positive relationship between the right inferior parietal lobe and inferior temporal gyrus and physical activity, while the control group demonstrated a negative relationship between those two regions. The false discovery rate corrected simple slopes demonstrated a significant relationship between the right inferior parietal lobule and the inferior temporal gyrus in the controls (Figure 5d). The interaction effect is less strong in the left inferior parietal lobe (Figure 5e).

## 4 DISCUSSION

Here we show that the functional connectivity between the dorsolateral prefrontal cortex and four clusters across the cortex exhibited differences in those with a TBI history compared to those with no TBI history. A general pattern of hypoconnectivity of long‐range cortico‐cortical connections and hyperconnectivity of short‐range cortico‐cortical connections was seen, where two posterior clusters (intracalcarine cortex and cerebellum) showed decreased connectivity in the TBI group, while the two anterior clusters (cingulate gyrus and frontal pole) showed increases in connectivity in the TBI group. Moreover, seed to voxel results revealed an association between physical activity and functional connectivity between the dorsolateral prefrontal cortex and the inferior temporal gyrus in those with a prior TBI, with greater physical activity associated with greater connectivity. The associations between physical activity and functional connectivity between the inferior parietal lobule and a distinct cluster within the inferior temporal gyrus was different in those with TBI compared to matched controls, with a positive relationship between functional connectivity and physical activity in TBI and a negative relationship in controls.

Overall, participants with a prior TBI showed hyperconnectivity in short‐range connections and hypoconnectivity in long‐range connections compared to age‐ and sex‐matched controls. These functional connectivity shifts may result from changes in firings of neurons, blood flow, oxidative metabolism, or a combination of these factors (Fox & Raichle, 2007; Mayer et al., 2011). Mechanistic processes that would affect the BOLD signal occur in TBI, such as reductions in cerebral perfusion (Soustiel & Sviri, 2007), increased reactivity of smooth muscle in the walls of vessels (Ueda et al., 2006), metabolic failure (Soustiel & Sviri, 2007), and decreases in the size of capillaries (Obenaus et al., 2017). These results are also supported by prior work showing FPCN connectivity differences between TBI and controls early after injury (DiFabio et al., 2022; Palacios et al., 2017). Interestingly, differences in functional connectivity in this study were seen in a cohort of participants regardless of age with varying times since injury, suggesting that these results may reflect a common change in the functional connectivity of the FPCN following a TBI with loss of consciousness.

The main aim of the study was to assess if engagement in physical activity was associated with functional connectivity of the FPCN in those with a TBI history. If so, physical activity interventions may then be promoted as a potential intervention for individuals living with the consequences of a TBI. We focused on the FPCN given the overlap of this network's involvement in TBI pathophysiology (Amir et al., 2021; Han et al., 2016) and physical exercise (Morris et al., 2022; Voss et al., 2010). No differences in physical activity were seen between groups, consistent with prior population‐based cohort studies (Morris et al., 2019), however, functional connectivity results demonstrate that (1) physical activity engagement is associated with the functional connectivity of the FPCN and (2) there are differences in how physical activity is associated with functional connectivity of the FPCN in those with a history and TBI compared to those without. A similar pattern of results has been reported previously, whereby despite a lack of difference in the proportion of individuals engaging in physical activity, physical activity was significantly and differentially associated with perceived cognitive health in those with a TBI history compared to those without (Morris et al., 2019). It is likely then that lifestyle interventions that aim to improve brain health in those with a TBI should be designed and tested within this specific population as differing results from healthy populations are possible. Notwithstanding, our results demonstrate that physical activity is associated with network‐level brain health in those with a history of TBI.

Our findings complement previous animal model literature in the field, adding to the understanding of how physical exercise (organized physical activity with the goal of improving physical fitness) can improve cognitive and brain functioning following a TBI (Griesbach et al., 2007; Karelina et al., 2021; Madathil & Saatman, 2015). Animal models have shown that the benefits of physical exercise after TBI are multifaceted with molecular, cellular, and network effects being shown (Griesbach et al., 2004, 2007; Sharma et al., 2020). Synaptic dysfunction is common after TBI and a driver of network changes after injury (Wolf & Koch, 2016). Our between group differences in functional connectivity of the FPCN show that a common signature of TBI exists in those with a history of TBI, which may give rise to the differential physical activity/functional connectivity relationship we see in this cohort (Wen et al., 2017). The within‐network functional connectivity between the dorsolateral prefrontal cortex and inferior temporal gyrus may be a potential mechanism through which physical activity can improve TBI‐related outcomes, albeit, interventional studies are needed to test this hypothesis. Indeed, prior work has demonstrated that physical activity is associated with the inferior temporal gyrus in non‐injured adults (Jacini et al., 2009; Raffin et al., 2023). Consequently, this may imply that physical activity interventions could be a way through which long‐term sustainable lifestyle interventions can promote brain health in those with a TBI.

There are several limitations to this study that need to be considered when interpreting the results. This study is purely cross‐sectional, and interventional studies are needed to definitively assess the mechanistic effects of physical activity on functional connectivity in TBI populations. The lack of a clinical characterization of TBI means the severity of injury in these participants is unknown and the self‐report nature of the diagnosis is open to reporting error. Nevertheless, the addition of reporting a loss of consciousness and the time since injury increases confidence in the accurate reporting of the condition. Physical activity definition is another limitation to consider, as this is a questionnaire based on recall and not objective. However, the questionnaire is extensive and addresses various types of physical activity. Further, biological sex is important to the recovery of TBI. Specifically, hormonal changes during menopause are associated with changes in functional connectivity (He et al., 2021) but are not addressed in this study. Furthermore, the age range of included participants is large. Age is included as a covariate to attempt to account for age‐related effects with reported results indicating an age‐independent effect of TBI of functional connectivity of the FPCN and the physical activity‐functional connectivity association. Additionally, this study only looks at physical activity and not other healthy lifestyle behaviors, such as sleep, diet, mental health, substance use, and others, which often cluster together (Ai et al., 2023). Future work should look to examine if there is a combined effect of broader lifestyle changes on functional connectivity within a TBI population.

## 5 CONCLUSIONS

Our results highlight the role physical activity may play in brain health following a TBI with loss of consciousness. Strategies to increase voluntary engagement in physical activity specifically in individuals living with a TBI are necessary, and beyond the well‐known general health benefits of physical activity, these strategies may also impact brain health in this population.

## AUTHOR CONTRIBUTIONS

**Emma M. Tinney**: Formal analysis; visualization; methodology; conceptualization; investigation; writing—original draft. **Meishan Ai**: Conceptualization; investigation; methodology; writing—review and editing; data curation; resources. **Goretti España‐Irla**: Writing—review and editing; conceptualization; methodology; software. **Charles H. Hillman**: Writing—review and editing; supervision; resources. **Timothy P. Morris**: Conceptualization; investigation; writing—review and editing; project administration; supervision; resources; validation.

### PEER REVIEW

The peer review history for this article is available at https://publons.com/publon/10.1002/brb3.70022.

## Data Availability

The data that support the findings of this study are openly available in Cam‐CAN at https://camcan‐archive.mrc‐cbu.cam.ac.uk/dataaccess/

## References

[brb370022-bib-0001] Ai, M. , Morris, T. P. , Zhang, J. , de la Colina, A. N. , Tremblay‐Mercier, J. , Villeneuve, S. , Whitfield‐Gabrieli, S. , Kramer, A. F. , & Geddes, M. R. (2023). Resting‐state MRI functional connectivity as a neural correlate of multidomain lifestyle adherence in older adults at risk for Alzheimer's disease. Scientific Reports, 13(1), Article 7487. 10.1038/s41598-023-32714-1 37160915 PMC10170147

[brb370022-bib-0002] Amir, J. , Nair, J. K. R. , Del Carpio‐O'Donovan, R. , Ptito, A. , Chen, J.‐K. , Chankowsky, J. , Tinawi, S. , Lunkova, E. , & Saluja, R. S. (2021). Atypical resting state functional connectivity in mild traumatic brain injury. Brain and Behavior, 11(8), e2261. 10.1002/brb3.2261 34152089 PMC8413771

[brb370022-bib-0003] Andersen, R. A. , & Cui, H. (2009). Intention, action planning, and decision making in parietal‐frontal circuits. Neuron, 63(5), 568–583. 10.1016/j.neuron.2009.08.028 19755101

[brb370022-bib-0004] Behzadi, Y. , Restom, K. , Liau, J. , & Liu, T. T. (2007). A component based noise correction method (CompCor) for BOLD and perfusion based fMRI. Neuroimage, 37(1), 90–101. 10.1016/j.neuroimage.2007.04.042 17560126 PMC2214855

[brb370022-bib-0005] Best, J. R. , Nagamatsu, L. S. , & Liu‐Ambrose, T. (2014). Improvements to executive function during exercise training predict maintenance of physical activity over the following year. Frontiers in Human Neuroscience, 8, 353. 10.3389/fnhum.2014.00353 24904387 PMC4034407

[brb370022-bib-0006] Brand, R. , & Ekkekakis, P. (2018). Affective–reflective theory of physical inactivity and exercise: Foundations and preliminary evidence. German Journal of Exercise and Sport Research, 48(1), 48–58. 10.1007/s12662-017-0477-9

[brb370022-bib-0007] Buckley, J. , Cohen, J. D. , Kramer, A. F. , McAuley, E. , & Mullen, S. P. (2014). Cognitive control in the self‐regulation of physical activity and sedentary behavior. Frontiers in Human Neuroscience, 8, 747. https://www.frontiersin.org/articles/10.3389/fnhum.2014.00747 25324754 10.3389/fnhum.2014.00747 PMC4179677

[brb370022-bib-0008] Cheval, B. , Orsholits, D. , Sieber, S. , Courvoisier, D. , Cullati, S. , & Boisgontier, M. P. (2020). Relationship between decline in cognitive resources and physical activity. Health Psychology, 39(6), 519–528. 10.1037/hea0000857 32202828

[brb370022-bib-0009] Churchill, N. , Hutchison, M. G. , Leung, G. , Graham, S. , & Schweizer, T. A. (2017). Changes in functional connectivity of the brain associated with a history of sport concussion: A preliminary investigation. Brain Injury, 31(1), 39–48. 10.1080/02699052.2016.1221135 27901587

[brb370022-bib-0010] Culham, J. C. , & Kanwisher, N. G. (2001). Neuroimaging of cognitive functions in human parietal cortex. Current Opinion in Neurobiology, 11(2), 157–163. 10.1016/s0959-4388(00)00191-4 11301234

[brb370022-bib-0011] Daly, M. , McMinn, D. , & Allan, J. L. (2015). A bidirectional relationship between physical activity and executive function in older adults. Frontiers in Human Neuroscience, 8, 112377. 10.3389/fnhum.2014.01044 PMC4292779 25628552

[brb370022-bib-0012] de Sousa Fernandes, M. S. , Ordônio, T. F. , Santos, G. C. J. , Santos, L. E. R. , Calazans, C. T. , Gomes, D. A. , & Santos, T. M. (2020). Effects of physical exercise on neuroplasticity and brain function: A systematic review in human and animal studies. Neural Plasticity, 2020, Article 8856621. 10.1155/2020/8856621 33414823 PMC7752270

[brb370022-bib-0013] DiFabio, M. S. , Smith, D. R. , Breedlove, K. M. , Pohlig, R. T. , Buckley, T. A. , & Johnson, C. L. (2022). Altered brain functional connectivity in the frontoparietal network following an ice hockey season. European Journal of Sport Science, 23, 684–692. 10.1080/17461391.2022.2069512 35466861

[brb370022-bib-0014] Dosenbach, N. U. F. , Fair, D. A. , Cohen, A. L. , Schlaggar, B. L. , & Petersen, S. E. (2008). A dual‐networks architecture of top‐down control. Trends in Cognitive Sciences, 12(3), 99–105. 10.1016/j.tics.2008.01.001 18262825 PMC3632449

[brb370022-bib-0015] Fox, M. D. , & Raichle, M. E. (2007). Spontaneous fluctuations in brain activity observed with functional magnetic resonance imaging. Nature Reviews Neuroscience, 8(9), 700–711. 10.1038/nrn2201 17704812

[brb370022-bib-0016] Geerligs, L. , Tsvetanov, K. A. , & Henson, R. N. , & Cam‐CAN . (2017). Challenges in measuring individual differences in functional connectivity using fMRI: The case of healthy aging. Human Brain Mapping, 38(8), 4125–4156. 10.1002/hbm.23653 28544076 PMC5518296

[brb370022-bib-0017] Gooijers, J. , Beets, I. A. M. , Albouy, G. , Beeckmans, K. , Michiels, K. , Sunaert, S. , & Swinnen, S. P. (2016). Movement preparation and execution: Differential functional activation patterns after traumatic brain injury. Brain, 139(9), 2469–2485. 10.1093/brain/aww177 27435093

[brb370022-bib-0018] Griesbach, G. S. , Gomez‐Pinilla, F. , & Hovda, D. A. (2004). The upregulation of plasticity‐related proteins following TBI is disrupted with acute voluntary exercise. Brain Research, 1016(2), 154–162. 10.1016/j.brainres.2004.04.079 15246851

[brb370022-bib-0019] Griesbach, G. S. , Gómez‐Pinilla, F. , & Hovda, D. A. (2007). Time window for voluntary exercise–induced increases in hippocampal neuroplasticity molecules after traumatic brain injury is severity dependent. Journal of Neurotrauma, 24(7), 1161–1171. 10.1089/neu.2006.0255 17610355

[brb370022-bib-0020] Hall, P. A. , & Fong, G. T. (2015). Temporal self‐regulation theory: A neurobiologically informed model for physical activity behavior. Frontiers in Human Neuroscience, 9, 117. https://www.frontiersin.org/articles/10.3389/fnhum.2015.00117 25859196 10.3389/fnhum.2015.00117 PMC4373277

[brb370022-bib-0021] Han, K. , Chapman, S. B. , & Krawczyk, D. C. (2016). Disrupted intrinsic connectivity among default, dorsal attention, and frontoparietal control networks in individuals with chronic traumatic brain injury. Journal of the International Neuropsychological Society, 22(2), 263–279. 10.1017/S1355617715001393 26888622 PMC4763346

[brb370022-bib-0022] He, L. , Guo, W. , Qiu, J. , An, X. , & Lu, W. (2021). Altered spontaneous brain activity in women during menopause transition and its association with cognitive function and serum estradiol level. Frontiers in Endocrinology, 12, 652512. https://www.frontiersin.org/articles/10.3389/fendo.2021.652512 34046011 10.3389/fendo.2021.652512 PMC8146460

[brb370022-bib-0023] Henry, C. J. K. (2005). Basal metabolic rate studies in humans: Measurement and development of new equations. Public Health Nutrition, 8(7a), 1133–1152. 10.1079/PHN2005801 16277825

[brb370022-bib-0024] Hofmann, W. , Schmeichel, B. J. , & Baddeley, A. D. (2012). Executive functions and self‐regulation. Trends in Cognitive Sciences, 16(3), 174–180. 10.1016/j.tics.2012.01.006 22336729

[brb370022-bib-0025] Jacini, W. F. S. , Cannonieri, G. C. , Fernandes, P. T. , Bonilha, L. , Cendes, F. , & Li, L. M. (2009). Can exercise shape your brain? Cortical differences associated with judo practice. Journal of Science and Medicine in Sport, 12(6), 688–690. 10.1016/j.jsams.2008.11.004 19147406

[brb370022-bib-0026] Karelina, K. , Schneiderman, K. , Shah, S. , Fitzgerald, J. , Cruz, R. V. , Oliverio, R. , Whitehead, B. , Yang, J. , & Weil, Z. M. (2021). Moderate intensity treadmill exercise increases survival of newborn hippocampal neurons and improves neurobehavioral outcomes after traumatic brain injury. Journal of Neurotrauma, 38(13), 1858–1869. 10.1089/neu.2020.7389 33470170 PMC8219196

[brb370022-bib-0027] Leddy, J. J. , Cox, J. L. , Baker, J. G. , Wack, D. S. , Pendergast, D. R. , Zivadinov, R. , & Willer, B. (2013). Exercise treatment for postconcussion syndrome: A pilot Study of changes in functional magnetic resonance imaging activation, physiology, and symptoms. The Journal of Head Trauma Rehabilitation, 28(4), 241. 10.1097/HTR.0b013e31826da964 23249769

[brb370022-bib-0028] Long, J. A. (2021). Interactions: Comprehensive, User‐Friendly Toolkit for Probing Interactions (Version 1.1.5) [Computer software]. CRAN. https://cran.r‐project.org/web/packages/interactions/index.html

[brb370022-bib-0029] Maas, A. I. R. , Menon, D. K. , Manley, G. T. , Abrams, M. , Åkerlund, C. , Andelic, N. , Aries, M. , Bashford, T. , Bell, M. J. , Bodien, Y. G. , Brett, B. L. , Büki, A. , Chesnut, R. M. , Citerio, G. , Clark, D. , Clasby, B. , Cooper, D. J. , Czeiter, E. , & Czosnyka, M. , … InTBIR Participants and Investigators . (2022). Traumatic brain injury: Progress and challenges in prevention, clinical care, and research. The Lancet Neurology, 21(11), 1004–1060. 10.1016/S1474-4422(22)00309-X 36183712 PMC10427240

[brb370022-bib-0030] Madathil, S. K. , & Saatman, K. E. (2015). IGF‐1/IGF‐R signaling in traumatic brain injury: Impact on cell survival, neurogenesis, and behavioral outcome. In F. H. Kobeissy (Ed.), Brain neurotrauma: Molecular, neuropsychological, and rehabilitation aspects. CRC Press/Taylor & Francis. http://www.ncbi.nlm.nih.gov/books/NBK299190/ 26269893

[brb370022-bib-0031] Mansouri, F. A. , Tanaka, K. , & Buckley, M. J. (2009). Conflict‐induced behavioural adjustment: A clue to the executive functions of the prefrontal cortex. Nature Reviews Neuroscience, 10(2), 141–152. 10.1038/nrn2538 19153577

[brb370022-bib-0032] Mayer, A. R. , Mannell, M. V. , Ling, J. , Gasparovic, C. , & Yeo, R. A. (2011). Functional connectivity in mild traumatic brain injury. Human Brain Mapping, 32(11), 1825–1835. 10.1002/hbm.21151 21259381 PMC3204375

[brb370022-bib-0033] Miller, E. K. , & Cohen, J. D. (2001). An integrative theory of prefrontal cortex function. Annual Review of Neuroscience, 24, 167–202. 10.1146/annurev.neuro.24.1.167 11283309

[brb370022-bib-0034] Miyake, A. , Friedman, N. P. , Emerson, M. J. , Witzki, A. H. , Howerter, A. , & Wager, T. D. (2000). The unity and diversity of executive functions and their contributions to complex “Frontal Lobe” tasks: A latent variable analysis. Cognitive Psychology, 41(1), 49–100. 10.1006/cogp.1999.0734 10945922

[brb370022-bib-0035] Moore, D. , Jung, M. , Hillman, C. H. , Kang, M. , & Loprinzi, P. D. (2022). Interrelationships between exercise, functional connectivity, and cognition among healthy adults: A systematic review. Psychophysiology, 59(6), e14014. 10.1111/psyp.14014 35122693

[brb370022-bib-0036] Morelli, N. , Johnson, N. F. , Kaiser, K. , Andreatta, R. D. , Heebner, N. R. , & Hoch, M. C. (2021). Resting state functional connectivity responses post‐mild traumatic brain injury: A systematic review. Brain Injury, 35(11), 1326–1337. 10.1080/02699052.2021.1972339 34487458

[brb370022-bib-0037] Morris, T. P. , Burzynska, A. , Voss, M. , Fanning, J. , Salerno, E. A. , Prakash, R. , Gothe, N. P. , Whitfield‐Gabrieli, S. , Hillman, C. H. , McAuley, E. , & Kramer, A. F. (2022). Brain structure and function predict adherence to an exercise intervention in older adults. Medicine and Science in Sports and Exercise, 54(9), 1483–1492. 10.1249/MSS.0000000000002949 35482769 PMC9378462

[brb370022-bib-0038] Morris, T. P. , Tormos Muñoz, J.‐M. , Cattaneo, G. , Solana‐Sánchez, J. , Bartrés‐Faz, D. , & Pascual‐Leone, A. (2019). Traumatic brain injury modifies the relationship between physical activity and global and cognitive health: Results from the barcelona brain health initiative. Frontiers in Behavioral Neuroscience, 13, 135. 10.3389/fnbeh.2019.00135 31275124 PMC6593392

[brb370022-bib-0039] Naumenko, Y. , Yuryshinetz, I. , Zabenko, Y. , & Pivneva, T. (2023). Mild traumatic brain injury as a pathological process. Heliyon, 9(7), e18342. 10.1016/j.heliyon.2023.e18342 37519712 PMC10372741

[brb370022-bib-0040] Nieto‐Castanon, A. (2020). Handbook of functional connectivity magnetic resonance imaging methods in CONN. Hilbert Press.

[brb370022-bib-0041] Obenaus, A. , Ng, M. , Orantes, A. M. , Kinney‐Lang, E. , Rashid, F. , Hamer, M. , DeFazio, R. A. , Tang, J. , Zhang, J. H. , & Pearce, W. J. (2017). Traumatic brain injury results in acute rarefication of the vascular network. Scientific Reports, 7(1), Article 239. 10.1038/s41598-017-00161-4 28331228 PMC5427893

[brb370022-bib-0042] Palacios, E. M. , Yuh, E. L. , Chang, Y.‐S. , Yue, J. K. , Schnyer, D. M. , Okonkwo, D. O. , Valadka, A. B. , Gordon, W. A. , Maas, A. I. R. , Vassar, M. , Manley, G. T. , & Mukherjee, P. (2017). Resting‐state functional connectivity alterations associated with six‐month outcomes in mild traumatic brain injury. Journal of Neurotrauma, 34(8), 1546–1557. 10.1089/neu.2016.4752 28085565 PMC5397233

[brb370022-bib-0043] Power, J. D. , Barnes, K. A. , Snyder, A. Z. , Schlaggar, B. L. , & Petersen, S. E. (2012). Spurious but systematic correlations in functional connectivity MRI networks arise from subject motion. Neuroimage, 59(3), 2142–2154. 10.1016/j.neuroimage.2011.10.018 22019881 PMC3254728

[brb370022-bib-0044] Puig, J. , Ellis, M. J. , Kornelsen, J. , Figley, T. D. , Figley, C. R. , Daunis‐i‐Estadella, P. , Mutch, W. A. C. , & Essig, M. (2020). Magnetic resonance imaging biomarkers of brain connectivity in predicting outcome after mild traumatic brain injury: A systematic review. Journal of Neurotrauma, 37(16), 1761–1776. 10.1089/neu.2019.6623 32228145

[brb370022-bib-0045] Raffin, J. , Rolland, Y. , Fischer, C. , Mangin, J.‐F. , Gabelle, A. , Vellas, B. , & de Souto Barreto, P. (2023). Cross‐sectional associations between cortical thickness and physical activity in older adults with spontaneous memory complaints: The MAPT Study. Journal of Sport and Health Science, 12(3), 324–332. 10.1016/j.jshs.2021.01.011 33545345 PMC10199140

[brb370022-bib-0046] Savikangas, T. , Törmäkangas, T. , Tirkkonen, A. , Alen, M. , Fielding, R. A. , Kivipelto, M. , Rantalainen, T. , Neely, A. S. , & Sipilä, S. (2021). The effects of a physical and cognitive training intervention vs. physical training alone on older adults’ physical activity: A randomized controlled trial with extended follow‐up during COVID‐19. PLoS One, 16(10), e0258559. 10.1371/journal.pone.0258559 34644357 PMC8513828

[brb370022-bib-0047] Seeley, W. W. , Menon, V. , Schatzberg, A. F. , Keller, J. , Glover, G. H. , Kenna, H. , Reiss, A. L. , & Greicius, M. D. (2007). Dissociable intrinsic connectivity networks for salience processing and executive control. Journal of Neuroscience, 27(9), 2349–2356. 10.1523/JNEUROSCI.5587-06.2007 17329432 PMC2680293

[brb370022-bib-0048] Shafto, M. A. , Tyler, L. K. , Dixon, M. , Taylor, J. R. , Rowe, J. B. , Cusack, R. , Calder, A. J. , Marslen‐Wilson, W. D. , Duncan, J. , Dalgleish, T. , Henson, R. N. , Brayne, C. , & Matthews, F. E. , & Cam‐CAN . (2014). The Cambridge Centre for ageing and neuroscience (Cam‐CAN) study protocol: A cross‐sectional, lifespan, multidisciplinary examination of healthy cognitive ageing. BMC Neurology, 14(1), 204. 10.1186/s12883-014-0204-1 25412575 PMC4219118

[brb370022-bib-0049] Sharma, B. , Allison, D. , Tucker, P. , Mabbott, D. , & Timmons, B. W. (2020). Cognitive and neural effects of exercise following traumatic brain injury: A systematic review of randomized and controlled clinical trials. Brain Injury, 34(2), 149–159. 10.1080/02699052.2019.1683892 31739694

[brb370022-bib-0050] Sharp, D. J. , Scott, G. , & Leech, R. (2014). Network dysfunction after traumatic brain injury. Nature Reviews Neurology, 10(3), 156–166. 10.1038/nrneurol.2014.15 24514870

[brb370022-bib-0051] Soustiel, J. F. , & Sviri, G. E. (2007). Monitoring of cerebral metabolism: Non‐ischemic impairment of oxidative metabolism following severe traumatic brain injury. Neurological Research, 29(7), 654–660. 10.1179/016164107x240017 18173902

[brb370022-bib-0052] Tanji, J. , & Hoshi, E. (2008). Role of the lateral prefrontal cortex in executive behavioral control. Physiological Reviews, 88(1), 37–57. 10.1152/physrev.00014.2007 18195082

[brb370022-bib-0053] Taylor, J. R. , Williams, N. , Cusack, R. , Auer, T. , Shafto, M. A. , Dixon, M. , Tyler, L. K. , Cam‐CAN ., & Henson, R. N. (2017). The Cambridge Centre for ageing and neuroscience (Cam‐CAN) data repository: Structural and functional MRI, MEG, and cognitive data from a cross‐sectional adult lifespan sample. Neuroimage, 144, 262–269. 10.1016/j.neuroimage.2015.09.018 26375206 PMC5182075

[brb370022-bib-0054] Ueda, Y. , Walker, S. A. , & Povlishock, J. T. (2006). Perivascular nerve damage in the cerebral circulation following traumatic brain injury. Acta Neuropathologica, 112(1), 85–94. 10.1007/s00401-005-0029-5 16718445

[brb370022-bib-0055] Vincent, J. L. , Kahn, I. , Snyder, A. Z. , Raichle, M. E. , & Buckner, R. L. (2008). Evidence for a frontoparietal control system revealed by intrinsic functional connectivity. Journal of Neurophysiology, 100(6), 3328–3342. 10.1152/jn.90355.2008 18799601 PMC2604839

[brb370022-bib-0056] Voss, M. , Prakash, R. , Erickson, K. , Basak, C. , Chaddock, L. , Kim, J. , Alves, H. , Heo, S. , Szabo, A. , White, S. , Wojcicki, T. , Mailey, E. , Gothe, N. , Olson, E. , McAuley, E. , & Kramer, A. (2010). Plasticity of brain networks in a randomized intervention trial of exercise training in older adults. Frontiers in Aging Neuroscience, 2, 1803. https://www.frontiersin.org/articles/10.3389/fnagi.2010.00032 10.3389/fnagi.2010.00032 PMC2947936 20890449

[brb370022-bib-0057] Wareham, N. J. , Jakes, R. W. , Rennie, K. L. , Mitchell, J. , Hennings, S. , & Day, N. E. (2002). Validity and repeatability of the EPIC‐Norfolk physical activity questionnaire. International Journal of Epidemiology, 31(1), 168–174. 10.1093/ije/31.1.168 11914316

[brb370022-bib-0058] Wen, Z. , Li, D. , Shen, M. , & Chen, G. (2017). Therapeutic potentials of synapses after traumatic brain injury: A comprehensive review. Neural Plasticity, 2017, e4296075. 10.1155/2017/4296075 PMC5405590 28491479

[brb370022-bib-0059] Whitfield‐Gabrieli, S. , & Nieto‐Castanon, A. (2012). Conn: A functional connectivity toolbox for correlated and anticorrelated brain networks. Brain Connectivity, 2(3), 125–141. 10.1089/brain.2012.0073 22642651

[brb370022-bib-0060] Wolf, J. A. , & Koch, P. F. (2016). Disruption of network synchrony and cognitive dysfunction after traumatic brain injury. Frontiers in Systems Neuroscience, 10, 43. 10.3389/fnsys.2016.00043 27242454 PMC4868948

[brb370022-bib-0061] Yu, Q. , Herold, F. , Becker, B. , Klugah‐Brown, B. , Zhang, Y. , Perrey, S. , Veronese, N. , Müller, N. G. , Kramer, A. F. , & Zou, L. (2021). Cognitive benefits of exercise interventions: An fMRI activation likelihood estimation meta‐analysis. Brain Structure and Function, 226(3), 601–619. 10.1007/s00429-021-02247-2 33675397

